# A Single Primary Blast-Induced Traumatic Brain Injury in a Rodent Model Causes Cell-Type Dependent Increase in Nicotinamide Adenine Dinucleotide Phosphate Oxidase Isoforms in Vulnerable Brain Regions

**DOI:** 10.1089/neu.2017.5358

**Published:** 2018-09-01

**Authors:** Kakulavarapu V. Rama Rao, Stephanie Iring, Daniel Younger, Matthew Kuriakose, Maciej Skotak, Eren Alay, Raj K. Gupta, Namas Chandra

**Affiliations:** ^1^ Center for Injury Biomechanics, Materials, and Medicine, Department of Biomedical Engineering, New Jersey Institute of Technology, Newark, New Jersey.; ^2^ Department of Defense Blast Injury Research Program Coordinating Office, United States Army Medical Research and Materiel Command, Fort Detrick, Maryland.

**Keywords:** astrocytes, blast injury, 4-hydroxynonenal, microglia, neuron, NADPH oxidase, oxidative stress, traumatic brain injury

## Abstract

Blast-induced traumatic brain injury (bTBI) is a leading cause of morbidity in soldiers on the battlefield and in training sites with long-term neurological and psychological pathologies. Previous studies from our laboratory demonstrated activation of oxidative stress pathways after blast injury, but their distribution among different brain regions and their impact on the pathogenesis of bTBI have not been explored. The present study examined the protein expression of two isoforms: nicotinamide adenine dinucleotide phosphate (NADPH) oxidase 1 and 2 (NOX1, NOX2), corresponding superoxide production, a downstream event of NOX activation, and the extent of lipid peroxidation adducts of 4-hydroxynonenal (4HNE) to a range of proteins. Brain injury was evaluated 4 h after the shock-wave exposure, and immunofluorescence signal quantification was performed in different brain regions. Expression of NOX isoforms displayed a differential increase in various brain regions: in hippocampus and thalamus, there was the highest increase of NOX1, whereas in the frontal cortex, there was the highest increase of NOX2 expression. Cell-specific analysis of changes in NOX expression with respect to corresponding controls revealed that blast resulted in a higher increase of NOX1 and NOX 2 levels in neurons compared with astrocytes and microglia. Blast exposure also resulted in increased superoxide levels in different brain regions, and such changes were reflected in 4HNE protein adduct formation. Collectively, this study demonstrates that primary blast TBI induces upregulation of NADPH oxidase isoforms in different regions of the brain parenchyma and that neurons appear to be at higher risk for oxidative damage compared with other neural cells.

## Introduction

Traumatic brain injury (TBI) resulting from different episodes of head trauma is one of the leading causes of morbidity and death in both military personnel and civilian populations. TBI causes approximately 1.5 million deaths and hospitalizations annually in the United States.^1–3^ Blast-induced TBI (bTBI) is the most prevalent form of brain injury in soldiers in combat zones because of the widespread use of high explosives in the war zones, and an increasing number of cases has also been reported in civilian populations with the use of improvised explosive devices by insurgents.^4,5^

Among many pathological factors associated with either primary mechanical injury or secondary biochemical cascades, oxidative stress has been shown to play a major role in various models of TBI.^6,7^ The main inducers of oxidative stress are reactive oxygen species (ROS), which include superoxide (O2⋅−), hydroxyl radical (HO⋅), and hydrogen peroxide (H_2_O_2_).^8,9^ ROS are normally produced in several metabolic reactions, including redox-reactions (oxidation/reduction), oxidative phosphorylation, and in a normal process of electron transport chain reactions. There are a number of enzymes that produce free radicals during their catalytic reactions, which include the nicotinamide adenine dinucleotide phosphate (NADPH) oxidase family, cytochrome P450 (CYP450), cyclooxygenase (COX), lipoxygenase (LOX), and xanthine oxidase.

The NADPH oxidase (NOX) is a multi-subunit enzyme that catalyzes the reduction of molecular oxygen and oxidation of NADPH to generate superoxide radicals (O_2_^•^−). NOX is comprised of subunits that are both plasma membrane-bound (cytochrome b_558_, composed of p22^phox^ and gp91^phox^) and cytoplasmic (p40^phox^, p47 ^phox^, and p67 ^phox^), which spans across the lipid bilayers.^10,11^ A number of NOX isoforms were identfied in the brain, which include NOX1, NOX2, and NOX4, and their cellular distribution is highly dependent on the cell type. Neurons express both NOX1 and NOX2, microglia are enriched with NOX2, while only small amounts of NOX isoforms were identified in astrocytes.^12^

Extensive experimental evidence suggests NOX plays a significant role in the pathophysiology of various forms of TBI. NOX has been shown to be upregulated in a brain in a controlled cortical impact model of trauma^13^ and closed head injury models.^14–16^ We have previously reported increased protein expression of NOX1 in a rodent model of a single blast injury at different blast overpressure exposures.^17,18^ While these studies establish a primary role of NOX1 in the pathophysiology of various forms of TBI, no studies have been performed to determine the spatial and temporal resolution of NOX family of enzymes in the brain and their role in the pathophysiology of bTBI. It is hypothesized that in bTBI, a single blast overpressure exposure is capable of biomechanically loading the whole brain, which may trigger a cascade of biochemical events consistent with regional vulnerabilities.

The present study therefore examined the spatial resolution of two isoforms of NOX (NOX1 and NOX2) and their cellular distribution and changes in rats exposed to moderate blast TBI. Levels of superoxide and formation of protein adducts of 4-hydroxynonenal (4HNE) were also determined. Based on the evidence that blast overpressure waves travel through the whole brain, we hypothesize that blast-induced NOX-related oxidative stress will be diffuse across the brain. Moreover, we hypothesize that different neural cell types have differential susceptibility to the oxidative stress-inducing effect of the primary blast.

## Methods

### Animals

Adult 10-week-old male Sprague-Dawley (Charles River Laboratories) rats weighing 320–360 g were used in all the studies. The animals were housed with free access to food and water in a 12-h dark-light cycle at 22°C. All procedures followed the guidelines established in the *Guide for the Care and Use of Laboratory Animals* and were approved by Rutgers University Institutional Animal Care and Use Committee before experiments. Rats were divided into two groups (sham controls and animals exposed to a moderate blast of 180 kPa).

A total number of 24 rats was used in this study as follows: immunoblotting (three controls and three blast injured); immunofluorescence studies (four controls and four blast-injured); superoxide production studies (five controls and five blast-injured). For immunofluorescence studies, each brain tissue was processed to obtain several sections (>10) from frontal cortex, striatum, hippocampus, thalamus, and cerebellum. Each of those sections were used for identification of NOX1 and NOX2 isoforms in neurons, astrocytes, and microglia by double immunofluorescence analysis. Similarly, for superoxide production, several sections from different brain regions of animals (five control and five blast-injured) were used for regional analyses.

### Blast injury

Rats were exposed to a single blast wave at the Center of Injury Biomechanics, Materials and Medicine (New Jersey Institute of Technology, Newark) in the 9-inch square cross section shock tube as described previously.^18,19^ The primary shockwave generated in this shock tube has been validated against the pressure-time profiles measured experimentally in the live-fire explosion experiments^10^ and against theoretical pressure-time profiles associated with the detonation of C4 explosive (see Kuriakose and colleagues^20^ for details).

**FIG. 1.**
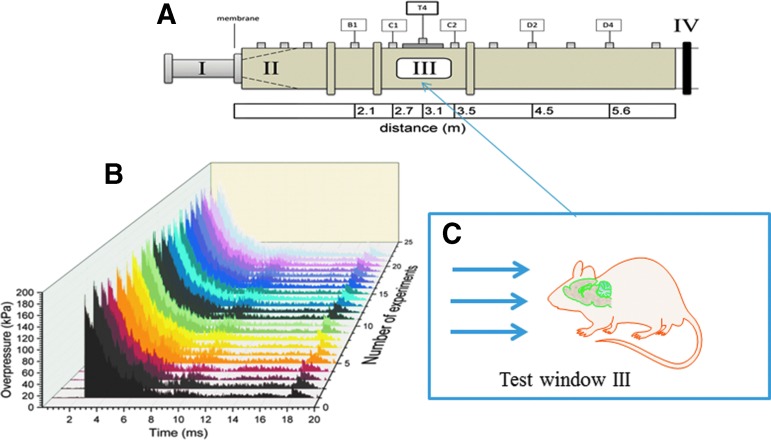
Schematic depiction of the shock tube. (**A**) Schematic of 9 × 9 inch square, 30 feet long shock tube with section I-Breech with high pressure helium gas separated from section II by different thickness of mylar sheets that generate pure shockwave in section III where the specimens are located. Section IV is past the section and is a design requirement; the pressure-time cycle is identical to live fire tests with actual C-4 (or trinitrotoluene equivalent) explosives at specified standoff distance. (**B**) Composite of actual experimental profiles that generate 180 kPa with only about five kPa variation in peak pressure and less than a millisecond in duration. The front of the pressure rise indicates shock-wave conditions. (**C**) Schematic of rodent model in prone position facing the shock front. The shock travels in the rostral-caudal direction traversing the pre-frontal cortex, striatum, hippocampus, thalamus, visual cortex, and cerebellum within a period of a millisecond with minimal attenuation of pressure loading. Color image is available online at www.liebertpub.com.neu

All rats were anesthetized with a mixture of ketamine (100 mg/kg) and xylazine (10 mg/kg) at 10:1 ratio administered via intraperitoneal injection. Rats underwent a single exposure to 180 ± 5 kPa peak overpressure (duration: 6.5 ± 0.5 msec, impulse: 320 ± 20 kPa·msec) and euthanized 4 h post-TBI. All rats were mounted in the middle of the shock tube (2.8 meters from the breech, and 3 meters from the exit) in a prone position—i.e. were strapped securely to the aluminum plate using a cotton cloth wrapped around the body (see Mishra and associates,^18^ Fig. 1B). The cloth provides no protection against the shockwave, but prevents any excessive head motion.^19^ Sham control rats received anesthesia and noise exposure but without blast exposure—i.e., anesthetized animals were placed next to the shock tube, and then a single blast was fired.

As a quality control measure, we have also monitored a high-speed video recording to capture any substantial head and body motion during the blast so as to exclude the impact of tertiary bTBI. After blast injury, animals were monitored closely for any signs of trauma-related distress (e.g., apnea). For immunoblot analysis, rats were anesthetized and transcardially perfused with phosphate buffered saline (PBS, pH 7.0) whereas for immunofluorescence studies, rats were first perfused with PBS followed by 4% paraformaldehyde (PFA); brains were then isolated and cryoprotected in 30% sucrose.

### Western blotting

Before evaluating spatial resolution of NOX1 and NOX2 protein changes in different brain regions by immunofluorescence, we first examined their levels in cerebral hemisphere and cerebellum by immunoblot. After perfusion with PBS, brains were excised from the cranial vaults, the whole left hemisphere and cerebellum were separately homogenized in ice-cold conditions using CellLytic-M (Sigma) using sonicator with probe amplitude set to 45%. Samples were then centrifuged at 14,000 × *g* at 4°C. The protein concentration in the samples was estimated by bicinchoninic acid (BCA) method (Thermo Scientific, Rockford, IL).

Subsequently, 10–20 μg of protein per lane was loaded into 4–15% SDS-PAGE gradient gels (Bio Rad). Proteins separated according to their molecular size were then transferred onto polyvinylidene difluoride (PVDF) membranes using Turbo Protein Transfer instrument (Bio Rad Laboratories) according to manufacturer instructions. Membranes were blocked with 5% milk dissolved in Tris-Buffered saline containing 0.1% Tween-20 (TBS-T) and incubated overnight at 4°C with NOX 1 antibody (Sigma-Aldrich) or NOX2 antibody (Novus Biologicals) or 4HNE (Abcam, Cambridge, MA) at a dilution of 1:1000. Bands were visualized using Western Pico Chemiluminescence Substrate (Thermo Scientific) on Chemi Doc Imaging System (Bio Rad Laboratories). For densitometric quantitation of Western blots, images were digitized using a BioRad GS800 calibrated densitometer, and analyzed with BioRad Quantity One software.

### Immunofluorescence and microscopy

To evaluate the spatial changes of NOX1 and NOX2 proteins in different brain regions, as well as to identify cell-specific changes in discrete brain regions, we performed double-immunofluorescence studies of two isoforms of NOX with NeuN, glial fibrillary acidic protein (GFAP) and Iba1, markers of neurons, astrocytes, and microglia, respectively, in frontal cortex, striatum, hippocampus, thalamus, and cerebellum. Briefly, 4 h post-injury, both sham and TBI animals were transcardially perfused with phosphate buffered saline (PBS) followed by 4% paraformaldehyde (PFA). After perfusion, the brains were removed from cranial vaults and incubated in 4% PFA for an additional 48 h and cryoprotected by immersing in 30% sucrose. Brains were then dissected into 2 mm thick sections using rat brain slicer (Kent Scientific Corp.) and embedded in OCT (Optimal Cutting Temperature) media and frozen quickly in isopentane cooled to liquid nitrogen temperature. Frozen sections were stored at −80°C until ready for sectioning.

Brain sections (20 μm thick) were prepared from the frozen tissue blocks, using Leica CM3050 cryostat, and immunofluorescence was performed. Briefly, tissue sections mounted on glass slides prepared from four individual animals in each group were washed with 10 mM PBS, fixed in ice-cold methanol (100%) solution for 10 min at −20°C. The tissue sections were blocked with 10% donkey serum at room temperature for 1 h in PBS containing 0.03% Triton X-100. Fixed tissues were incubated overnight at 4°C with respective primary antibodies to NOX1, NOX2, GFAP, NeuN, and Iba1. The details on the source, clonality, and the dilution of each antibody used are provided in Table 1.

**Table 1.** Antibodies Used in the Current Study

*Antibody*	*Source*	*Catalog number*	*Host-species*	*Dilution*
NADPH oxidase 1	Sigma-Aldrich Inc.	SAB420097-200UL	Rabbit (polyclonal)	1:400
NADPH oxidase 2	Invitrogen Inc.	PA5-34600	Rabbit (Polyclonal)	1:250
Glial fibrillary acidic protein (GFAP)	ThermoFisher Scientific Inc.	MA5-12023	Mouse (Monoclonal)	1:400
NeuN	Abcam	AB104224	Mouse (Monoclonal)	1:400
Iba1	Invitrogen Inc.	PA5-18039	Goat (Polyclonal)	1:250

NADPH, nicotinamide adenine dinucleotide phosphate.

Double immunofluorescence was performed using donkey-antirabbit Alexafluor 594 for NOX1 or NOX2 and donkey-antimouse Alexafluor 488 for GFAP, donkey-antirabbit Alexafluor 488 for NeuN, and donkey-antigoat Alexafluor 488 for Iba1. The specificity of each antibody staining was validated by excluding each primary antibody (negative controls) and visualized for any nonspecific fluorescence. The primary antibody specificity, however, was not validated independently by blocking the binding to tissue with the corresponding antigen. Slides containing different brain regions were digitized (20x magnification) using Leica Aperio Versa 200 fluorescent microscope and slide scanner. Fluorescence intensities in each region were quantitated using AreaQuant software (Leica Biosystems) and expressed as average fluorescence intensity/unit area.

### Superoxide production

Superoxide (O_2._^-^) levels in different brain regions were measured using dihydroethidium (DHE) following the method of Kim and coworkers.^21^ Briefly, control and blast-induced animals (five controls and five animals immediately after blast) were injected with 5 mg/kg DHE (Molecular Probes, MA), dissolved in dimethylsulfoxide (DMSO) intraperitoneally, and 4 h after blast, animals were transcardially perfused first with PBS followed by 4% PFA, brains excised, and 50 μm thin sections of different brain regions were prepared using Leica VT 1000S vibratome and mounted. DHE immunofluorescence in each region was visualized by digitizing the images using Leica Aperio Versa 200 slide scanner. Fluorescent intensities in each region were quantitated using AreaQuant software (Leica Biosystems) and expressed as average fluorescence intensity/unit area.

### Image acquisition and analysis

Slides with mounted coronal sections from the brain were imaged at 20x magnification using a Leica Aperio Versa 200 digital pathology scanner. Control sections were used as reference for adjusting the exposure times and gray scale balance for optimal image quality; once set, these parameters were fixed and used for image acquisition of the reminder of both control and experimental groups. Three channels were collected for each coronal section. Blue: 405 nm (DAPI), red: 594 nm (NOX1), and green: 488 nm (cell specific marker GFAP [astrocytes], Iba1 [microglia], and NeuN [neurons]). We then manually outlined the regions of interest in different brain structures, and the fluorescence intensities in each brain region were quantitated using FLAreaQuantV1 algorithm (Leica Biosystems) and expressed as average fluorescence intensity/unit stained area.

For each channel, we set a minimum intensity threshold value using control sections as reference that will exclude any background fluorescence caused by nonspecific binding of fluorescent secondary antibody, and the same threshold values were used to quantify both control and experimental groups. A maximum intensity threshold was also set to remove any oversaturation from excess fluorescent dye. The algorithm outputs the area of positive staining for each brain region, the average intensity of each channel, and intensity profile of each protein.

### Statistical analysis

Data are presented as mean ± standard error of the mean (SEM). Between-group comparisons were made by one-way analysis of variance (ANOVA) with a *post hoc* test (Bonferroni) to determine individual group differences. Differences between means were assessed at the probability level of *p* ≤ 0.05, 0.01, and 0.001. GraphPad Prism 6.0 software was used in all analyses and preparation of plots.

## Results

### Moderate blast over pressure increases the protein levels of NOX1 and NOX2

**FIG. 2.**
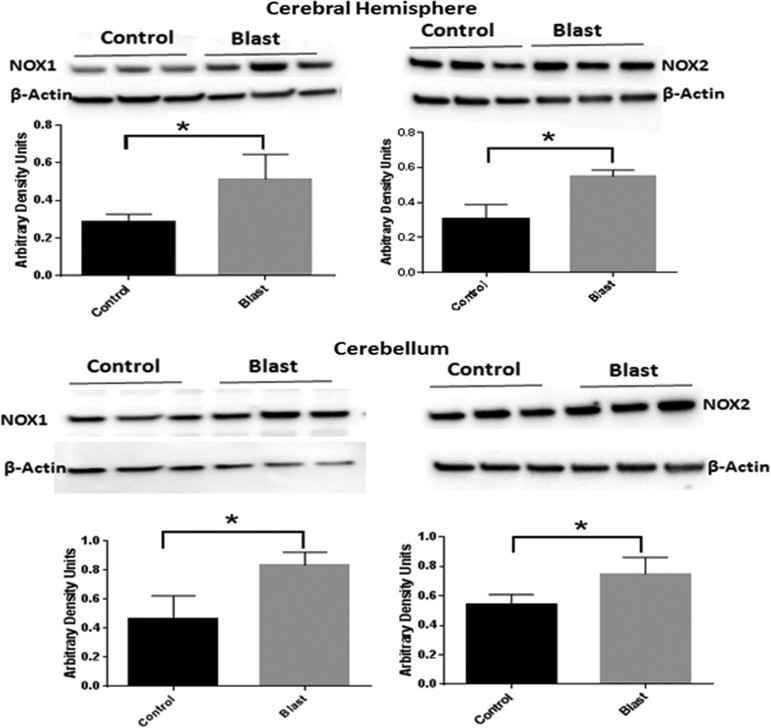
Blast increases the expression of nicotinamide adenine dinucleotide phosphate oxidase (NOX) isoforms. Immunoblots of NOX1 and NOX2 isoforms in the cerebral hemisphere and cerebellum 4 h after blast at 180 kPa blast over pressure. There was a significant increase (80%) in NOX1 in both cerebral hemispheres and cerebellum, while NOX2 increased by 83% in cerebral hemispheres and 38% in cerebellum. *n* = 3, *p < 0.05.

Previous studies in this laboratory identified increased oxidative and nitrosative stress factors in the cerebral cortex in rats exposed to mild bTBI.^17,18^ This study evaluated further the effect of moderate blast (180 kPa peak overpressure) on the early evolution of the protein expression of NOX isoforms (NOX1 and NOX2). Immunoblot analysis of NOX1 and NOX2 in the whole cerebral hemisphere showed a significant increase (87% and 52%, respectively, *p* < 0.05) (Fig. 2). To assess the diffuse nature of primary blast (shockwave) in the posterior region of the brain, NOX1 and NOX2 protein levels were also determined in the cerebellum, and we found that similar to cerebral hemisphere, cerebellar levels of NOX1and NOX2 protein were significantly increased (60% and 40%, respectively, *p* < 0.05) (Fig. 2).

### Differential changes in NOX isoforms expression in different brain regions

We next examined regional variations in NOX1 and NOX2 protein levels in the following brain structures: frontal cortex, striatum, hippocampus, thalamus, and cerebellum. The rationale in examining these regions is as follows: (a) NOX1 and NOX 2 isoforms are ubiquitously expressed in all brain regions, (b) the effect of shockwave propagation over the entire brain is not known, and (c) to use NOX1 and NOX2 as markers to evaluate whether there exists any selective vulnerability of various brain structures, a phenomenon that has not been investigated previously.

**FIG. 3.**
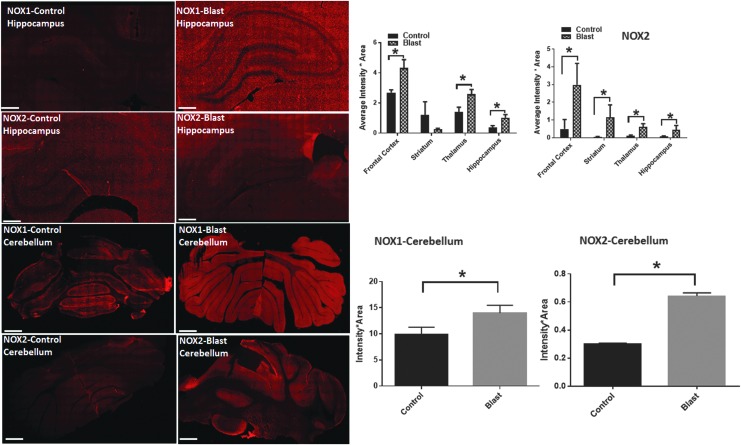
Nicotinamide adenine dinucleotide phosphate oxidase (NOX) isoforms show a differential increase in different brain regions. Fluorescence intensities (red) of NOX1 and NOX2 in the hippocampus and cerebellum from control and blast-injury animals. Quantification of fluorescence intensities in different brain regions show a striking increase in NOX1 in the hippocampus of blast-injured animals compared with controls. Intensities of NOX2 display a higher increase in the frontal cortex compared with other regions. *n* = 4. *p < 0.01–0.05. Fluorescent intensities NOX1 in the cerebellum display a striking increase (96%) compared with NOX2 (38%). Color image is available online at www.liebertpub.com.neu

Under primary blast loading conditions, the pathological changes were found throughout the brain as indicated by changes in the fluorescent intensities of NOX1 and NOX 2 in different brain regions. Interestingly. various regions displayed a differential response. Thus, NOX1 levels in the frontal cortex showed a 49% increase; hippocampus showed the highest degree of increase (107%) followed by thalamus displaying a 90% increase (Fig. 3). Total NOX1 levels did not change in the striatum and somatosensory barrel cortex (S1BF) (data not shown).

The regional variations in the levels of NOX2 are slightly different from that of NOX1. Total NOX2 levels were highest in the frontal cortex (>two-fold) followed by the striatum and hippocampus that showed the lowest increase (Fig. 3). NOX isoform expression was also increased in other discrete brain regions from rostral and caudal areas, among which theCA1 region of a hippocampus displayed the biggest increase (Table 2).

**Table 2.** Protein Levels of Nicotinamide Adenine Dinucleotide Phosphate Oxidase Isoform Expression in Different Brain Regions (from Rostral to Caudal Areas) in Control and Animals Four Hours after Exposed to Moderate Blast (180 kPa)

*Region*	*NOX 1 (% over Control)*	*NOX2 (% over Control)*
Hippocampus CA1	411%	+142%
Hippocampus CA3	265%	+203%
Dentate gyrus	605%	+20%
Corpus callosum	−77%	−65%
Cingulate cortex	+0.4%	−12.8%
Amygdala	+89%	+51.8%
Hypothalamus	+69%	+33.13

NOX, nicotinamide adenne dincleotide phosphate.

Note that highest increase in NOX expression was found in the CA1 region of the hippocampus that correlates with the known vulnerability to oxidative damage.

### Different neural cell types display differential vulnerability to oxidative damage

To evaluate cellular vulnerability to oxidative damage resulting from primary blast, changes in NOX1 expression patterns in astrocytes, neurons, and microglia were determined by double immunofluorescence staining. In addition, simultaneous quantification of NOX immunofluorescence using cell-specific markers will provide an insight into changes in its expression in different neural cells including astrocytes, neurons, and microglia.

**FIG. 4.**
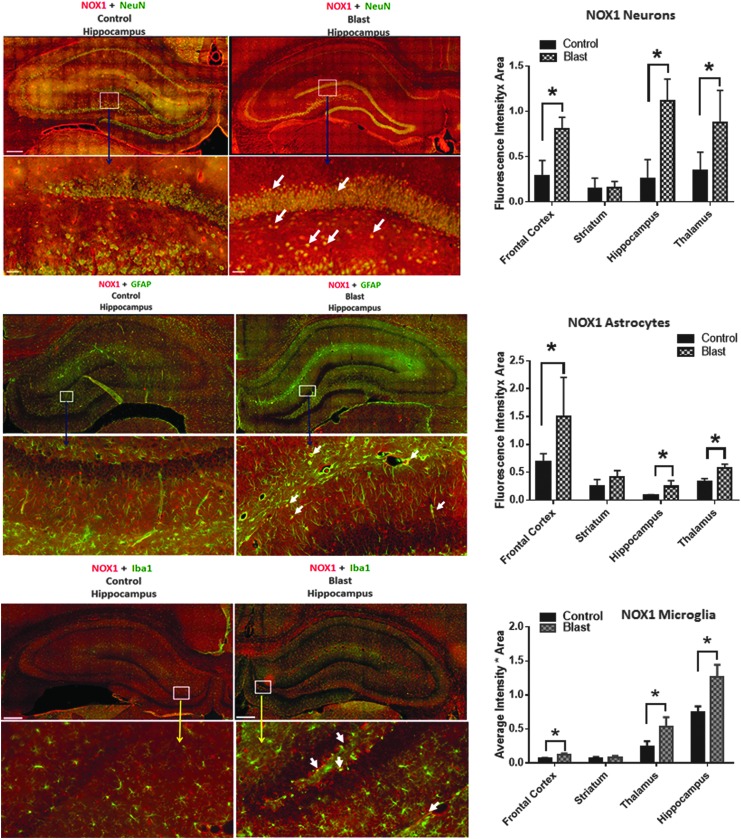
Nicotinamide adenine dinucleotide phosphate oxidase (NOX)1 shows greater co-localization in neurons. Representative merged images showing the co-localization of NOX1 with NeuN, GFAP and Iba1 in the hippocampus indicating neuronal, astrocytic, and microglia localization, respectively, of NOX1 in control and blast-injured animals. Majority of increase in NOX1 with respect to corresponding controls is in neurons compared with astrocytes and microglia. Quantification of florescence intensities in different brain regions show a striking increase in NOX1 fluorescence in neurons in the hippocampus of blast-injured animals compared with controls. *n* = 4, *p < 0.01–0.05. Color image is available online at www.liebertpub.com.neu

**FIG. 5.**
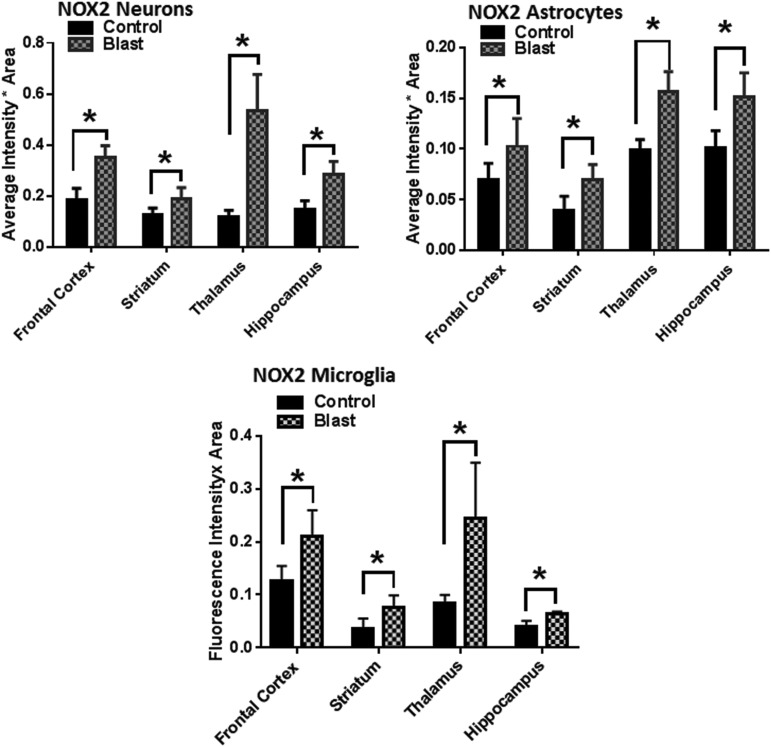
Neurons show the highest increase in nicotinamide adenine dinucleotide phosphate oxidase (NOX)2 expression. Quantification of florescence intensities in different brain regions show a striking increase in NOX2 fluorescence in the hippocampus and thalamus of blast-injured animals compared with other brain regions. *n* = 4, *p < 0.01–0.05.

Cell-specific analysis of increase in NOX1 and NOX2 levels after bTBI compared with their baseline expression (controls) indicate that neurons display a higher increase compared with astrocytes and microglia. In addition, NOX1 and NOX2 increases were more pronounced in neurons in the hippocampus and thalamus, respectively, compared with the frontal cortex. These conclusions were deduced based on the double immunofluorescence analysis showing co-localization of NOX1 with NeuN (a marker protein for neurons), with GFAP (a marker for astrocytes) and with Iba1 (a marker for microglia). (Fig. 4 and 5).

### Primary blast also significantly affects posterior brain structures including the cerebellum

**FIG. 6.**
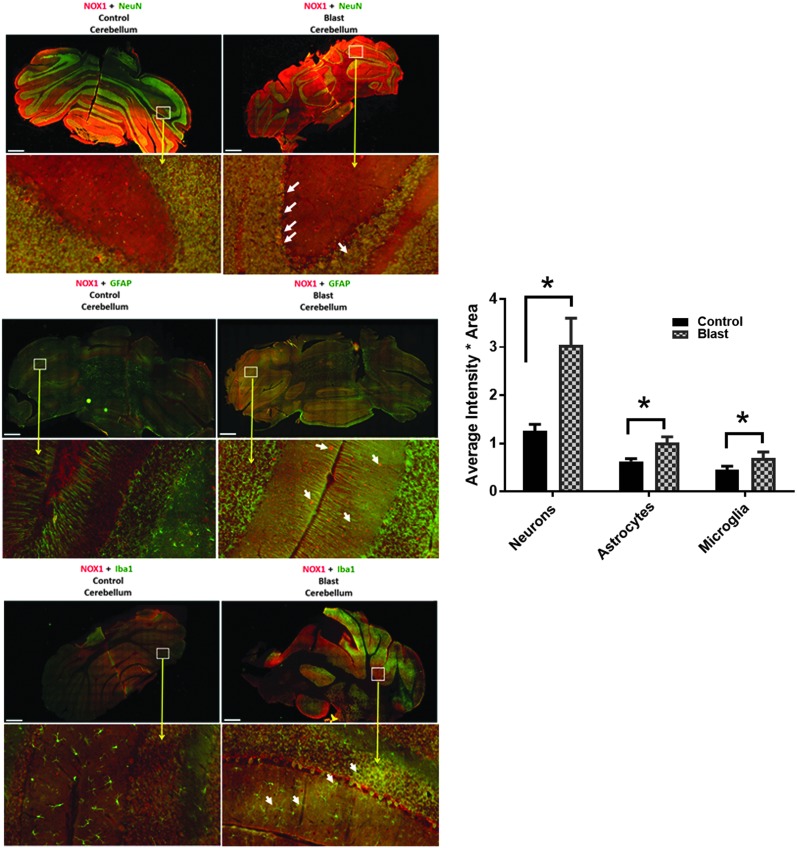
Cerebellum displays an increased nicotinamide adenine dinucleotide phosphate oxidase (NOX)1 expression in neurons compared with astrocytes and microglia. Representative merged images showing the co-localization of NOX1 with NeuN, GFAP, and Iba1 in the cerebellum indicating neuronal, astrocytic, and microglia localization, respectively, of NOX1 in control and blast-injured animals. *n* = 4, *p < 0.01–0.05. Color image is available online at www.liebertpub.com.neu

**FIG. 7.**
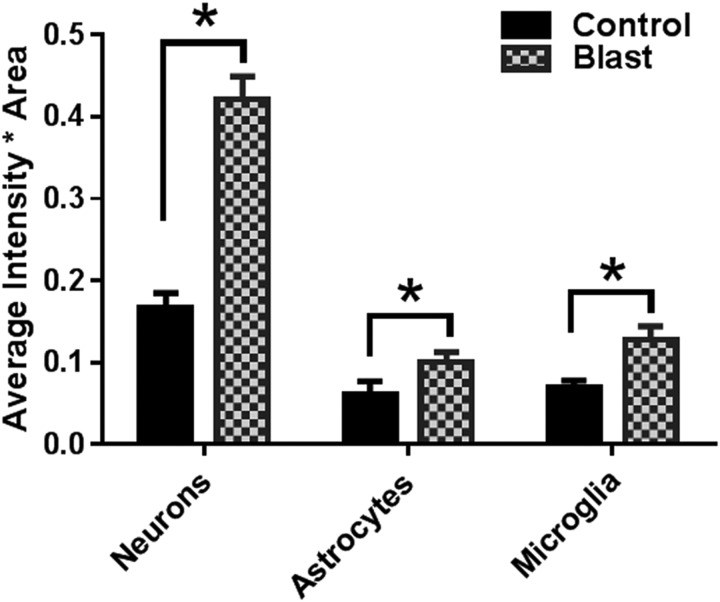
Nicotinamide adenine dinucleotide phosphate oxidase-2 (NOX)2 displays a greater increase in neurons compared with astrocytes and microglia. Similar to NOX1, the majority of NOX2 is localized in neurons compared with astrocytes and microglia. n = 4, *p < 0.01–0.05.

Primary blast caused diffused pathological changes not only in the perpendicular direction (deeper brain structures, hippocampus, thalamus) but also in the parallel direction to the wave propagation from the pre-frontal cortex to the cerebellum. The total tissue levels of NOX1 in the cerebellum not only increased, but this increase was highest in neurons (Fig. 6). Such higher expression of NOX1 in neurons compared with other neural cells also correlates with the known fact that the cerebellum contains the highest density of neurons compared with other brain regions.^22,23^ Similar to NOX1, elevated levels of NOX2 expression were also found to be mainly co-localized with neurons (Fig. 7).

### Primary blast increases superoxide levels in different brain regions

**FIG. 8.**
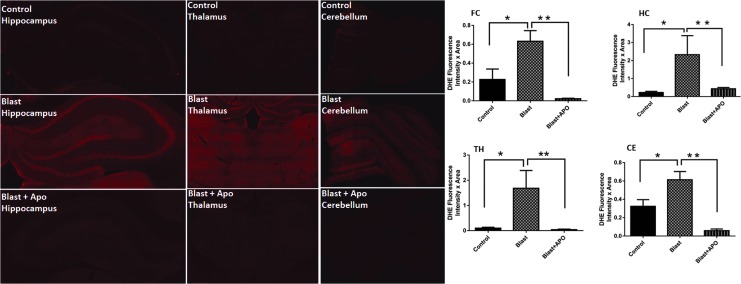
Primary blast increases superoxide levels in different brain regions. Representative fluorescent intensities (red) of dihydroethidium (DHE, dye that recognizes superoxide production) in hippocampus, thalamus and cerebellum in control, Blast-injured and blast+ apocynin. Quantification of fluorescence intensities in different brain regions shows a striking increase in DHE fluorescence in the hippocampus of blast-injured animals compared with controls indicating high levels of superoxide production in the hippocampus. Note that a pre-treatment with apocynin (APO), an inhibitor of nicotinamide adenine dinucleotide phosphate oxidase (NOX) activation, completely blocks the DHE fluorescence increase indicating that the superoxide increase is mediated by activation of NOX. *n* = 5 *p < 0.01–0.05. Color image is available online at www.liebertpub.com.neu

The activation of a variety of NOX isoforms is usually associated with the increased production of superoxide.^12,24^ Because the present study showed increased protein levels of NOX isoforms, we then sought to examine whether increased NOX protein after blast has a functional significance. Accordingly, *in vivo* levels of superoxide in the frontal cortex, hippocampus, thalamus, and cerebellum were measured using DHE. Hippocampus displayed a robust level of the increase in superoxide (>10 fold, *p* < 0.001) followed by thalamus and frontal cortex. It is noteworthy that the extent of the rise in superoxide production in the hippocampus and thalamus (Fig. 8) correlated well with the increased degree of NOX expression. In addition, a pre-treatment of animals with apocynin, which is known to block the assembly of different NOX subunits, completely inhibited the increase in superoxide production indicating an essential role of NOX in brain superoxide production after moderate blast.

### Primary blast results in oxidative damage and lipid peroxidation products in different brain structures

Several reports indicate the TBI resulting from different etiologies, including primary blast and blunt injuries, display oxidative stress during the evolution of its symptoms.^7,25–27^ Accordingly, several pathways are involved directly in the induction of oxidative stress including the activation of NOX^15,28–30^ and alterations of antioxidant defense mechanisms (a reduction in superoxide dismutase, catalase, glutathione peroxidase).^9,31^

One of the major downstream effects of oxidative stress in many neurological disorders and TBI is the formation and accumulation of lipid peroxidation products.^32–35^ Lipid peroxidation is a process under which oxidants such as free radicals attack various lipids containing carbon double bonds, and the resulting aldehyde products such as 4HNE generated by the lipid peroxidation directly modify amino acid structures in proteins and form adducts.^36^

**FIG. 9.**
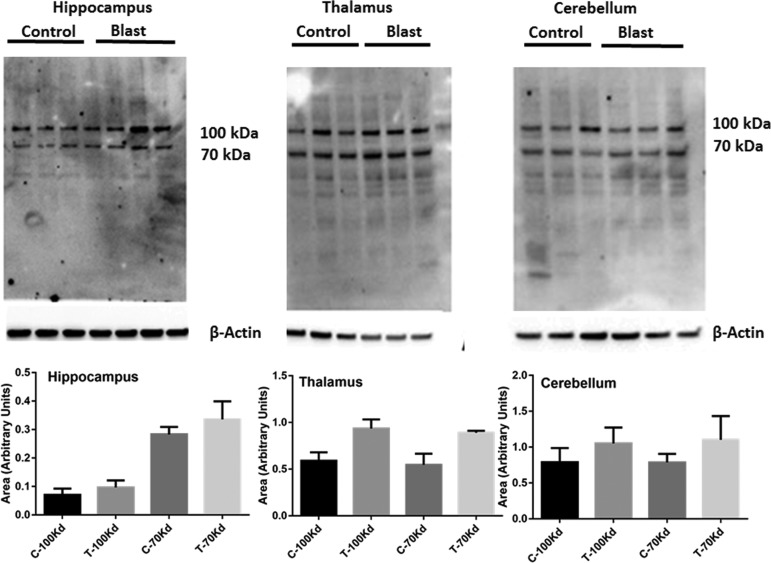
Primary blast displays a strong tendency to increase 4HNE protein adducts. Immunoblot analysis of 4HNE in lysates from the hippocampus, thalamus, and cerebellum 4 h after blast at 180 kPa blast overpressure is shown. Two proteins of 100 and 70 kDa show a strong tendency to increase in the hippocampus and thalamus. *n* = 3.

Superoxide is one of the free radicals that can directly oxidize lipids to aldehydes that leads to the formation of protein adducts and activation of NOX produces excess levels of superoxide.^25,37,38^ Because the present study clearly showed an increase in NOX proteins and increased levels of superoxide after bTBI, we sought to examine the levels of 4HNE adducts in discrete brain regions in rats subjected to moderate blast (180 kPa, 4 h). Immunoblot analysis of 4HNE products identified two major bands corresponding to molecular weights of 70 and 100 kDa, which showed a trend toward an increase in cerebral hemispheres, hippocampus, thalamus, and cerebellum; however, such changes were not significant (Fig. 9).

## Discussion

This study demonstrates that protein levels of superoxide producing enzymes NOX1 and NOX2 were significantly increased to different levels in various brain regions in rats exposed to moderate primary blast overpressure. Increased NOX expression was accompanied by increased superoxide production and a strong tendency toward increased HNE adduct formation in two proteins. Together these observations strongly indicate that there is an increase in oxidative stress and oxidative damage occurring in the early phase after a single exposure to a shockwave. This work is motivated by two related questions: (1) does the blast TBI affect different brain regions equally assuming homogeneous spatial distribution of biomechanical loading, and (2) does the extent of the damage vary between different brain regions containing disparate ratios of neural cells (neurons, astrocytes, and microglia)? Hence, we focused on the role of two different forms of superoxide producing enzymes and their downstream oxidative damage markers to understand both the spatial and cellular effects.

The bTBI has an unique pattern of biomechanical phenotype in that the external forces are dictated by the size and shape of the interacting body—in this instance, skull and brain structures. Further, the magnitude of forces generated depends on the contact area and interaction time that in turn determine the overall biomechanical loads at the tissue level. This loading triggers secondary biochemical cascades depending on the local tissue content. The cascade of secondary events usually develops over a period of hours to days that ultimately lead to additional neuropathological sequela.^39–41^ Among many causal factors implicated in the pathophysiology of bTBI, oxidative stress represents an important early pathological outcome resulting from either the activation of free radical producing enzymes or downregulation of antioxidant defense mechanisms.^7,26,27,42^

NOX is a superoxide producing enzyme and different isoforms of NOX, including NOX1, NOX2, and NOX4 have been identified in the brain.^12^ Studies reported increased activation of different isoforms of NOX in various forms of TBI. Accordingly, in a mouse model of controlled cortical impact (CCI), increased NOX2 expression was observed at 24–48 h after injury^43^ whereas Byrnes and associates^44^ found a delayed increase in NOX2 activity one month post-injury. In a rat model of CCI, however, increased NOX activity was observed as early as 1 h post-injury.^44^ Increased NOX expression was also found in different animal models of fluid percussion injury.^14.45.46^ In addition, apocynin, an inhibitor of NOX activation, showed protective effects on various models of blunt TBI.^16,46^ Together these studies highlight the critical role of NOX in the pathology of TBI.

While studies have established the importance of NOX in the pathophysiology of blunt TBI, there are limited studies of NOX in bTBI.^47–49^ This could in part be because of the lack of a true understanding the role of biomechanical loading in blast injury from limited availability of a field validated shock tube that simulates battlefield injuries. In the present study, we therefore characterized the spatial resolution of two isoforms of NOX (NOX1 and NOX2) in various brain regions and their localization in neurons, astrocytes, and microglia 4 h after blast injury in a field validated shock tube.^10,19,20^

A generalized increase in NOX protein levels by immunoblots in the present study is comparable to our previous reports indicating its upregulation in the homogenates of a cerebral cortex in rats exposed to different blast overpressures.^17,18^ Further, using immunofluorescence analysis, the present study identified regional variations in the expression pattern of NOX 1 in that the highest increase is found in the hippocampus followed by the thalamus. Interestingly, the frontal cortex showed higher expression in NOX2 compared with that in the hippocampus and thalamus. Other fine brain structures, including motor cortex, amygdala, and hypothalamus, also showed a significant increase in both the isoforms of NOX with the exception that NOX2 levels were significantly lower in a motor cortex (Table 2).

Interestingly, in the present study, a single blast exposure also affected distal brain structures, including the cerebellum, suggesting that blast injury propagation is highly diffusive in nature. While it is interesting to observe differential vulnerability of various brain structures to blast injury, in blast, a high-velocity shockwave traverses across and through the entire body loading all the regions almost simultaneously within a matter for 5–10 milliseconds. Recent experimental evidence in animal models and human cadeveric heads has shown that the shockwaves are capable of passing through the skin and skull and load all the brain structures.^10^

In addition to regional variations observed in NOX levels, we also found a cellular heterogeneity in the expression of NOX isoforms. Thus, blast injury displayed a robust increase in the levels of both isoforms of NOX in neurons compared to astrocytes and microglia with respect to their baseline values (controls). Studies have shown that all neural cells express different isoforms of NOX, including NOX1, NOX2, and NOX4.^50^ In addition, within the neurons, NOX1 is abundantly expressed in cerebellar granule cells,^51^ while NOX2 has been shown to be expressed in both cerebellar granule cells as well as hippocampal neurons.^52^ In addition to NOX1, the present study found a striking increase in NOX2 in neurons of the hippocampus, thalamus, and cerebellum compared with microglia.

Our results are in slight contrast to other reports wherein NOX2 is abundantly expressed in microglia.^44,53–55^ While the reason for apparent abundance of NOX2 in neurons in our study is not known, studies report that NOX2 upregulation in hippocampal pyramidal neurons drives neuropathology associated with psychosocial stress in rats.^56,57^ Further, studies also report that cellular injury to neurons after stroke resulted in an early upregulation of NOX2 in neurons (3–6 h after injury) whereas NOX2 was upregulated in microglia at later time points (72 h post-injury).^58,59^ Such temporal difference in the expression pattern of NOX2 between neurons and microglia is reasonable because neurons may be far more vulnerable acutely in the evolution of the injury process whereas microglial activation could be a relatively late phenomenon.

Superoxide is a major free radical produced in the brain by a variety of reactions, including disturbances in mitochondrial oxidative phosphorylation, increased production of arachidonic acid as a consequence of activation of phosphilipase A2 (PLA2), activation of xanthine oxidase as well as by the activation of NOX.^60–63^ Increased superoxide production has been shown in different models of TBI.^64–67^ The present study observed a striking increase in superoxide production in the hippocampus, thalamus, and frontal cortex, areas where corresponding increases in NOX levels were found. Moreover, our studies showing a complete absence of O_2_^•^− in animals treated with apocynin (an inhibitor of NOX activation) before the exposure to blast injury reinforces the finding that the increase in O_2_^•^− is indeed mediated by NOX activation. Our results are also in agreement with previous studies showing protective effects of apocynin in attenuating oxidative damage in different models of TBI.^15,16,30,47,68,69^

While in the present study, the superoxide levels were found to be strikingly increased in different brain regions, the levels of 4HNE adducts showed a trend toward the increase in two proteins with average molecular weights of 70 and 100 kDa, but did not show statistically significant changes. The reason behind the lack of significant changes is not known. The 4HNE products, however, can be formed via peroxidation of unsaturated fatty acids not only by superoxide radical but also by a variety of other reactive oxygen species, including hydroxyl, peroxyl as well as a varety of cyclic compounds.^70^ It is therefore likely that lipid peroxidation reaction and subsequent 4HNE formation may not have achieved a threshold as to show significant changes at 4 h after blast because reactive oxygen species other than superoxide (e.g., hydroxyl radicals among others) may not have been increased in the early phase of blast injury.

Despite an observed increase in factors conducive to oxidative damage in neurons, in the present study, we did not observe neurodegeneration nor apoptotic cell death as investigated by fluorescent staining for Fluorojade C and cleaved caspase-3 4 h after blast injury (data not shown). Perhaps a 4 h window of blast injury may be too early to detect neuronal death. It is also likely that moderate blast may not cause neuronal death because of the diffuse nature of the shockwave unlike blunt injuries where neuronal death was observed close to the epicenter in the acute phase of injury.^71^

The selective vulnerability of neurons to oxidative damage may be dictated not only by the propagation of primary shockwave, the so-called direct mechanical impact throughout the brain regions, but also in part influenced by the cellular density and cell types distribution in different brain regions. Recently, it has been shown that the cerebellum and hippocampus contain the highest density of neurons compared with a cerebral cortex although relative mass of the hippocampus and cerebellum is far lesser than cerebral cortex.^23,72^ In the present study, our observation of the highest increase in NOX levels in neurons in the hippocampus and cerebellum after blast suggests that neuron rich regions may be at higher risk for oxidative damage than other brain regions. This is further supported by our observation of a striking increase in superoxide levels in the hippocampus after blast. Also, within the hippocampus, studies have shown that neurons in CA1 region are more vulnerable to superoxide than CA3 neurons.^73,74^

Moreover, it is interesting to note that NOX1 showed a greater increase in CA1 region compared with CA3 (Table 2). Such higher level of neuronal oxidative damage in discrete brain regions may also depend on a number of other factors, including vascular density and associated metabolic supply and consumption of glucose and oxygen, neuronal excitability, and synaptic transmission.^75^ In support of this tenet, it has been shown that the density (and perhaps subsequent nutrient supply) of brain capillaries was far less in the CA1 region of the hippocampus compared with the CA3 region, which likely places the CA1 region at higher risk for ischemia and hypoxia.^76^

**FIG. 10.**
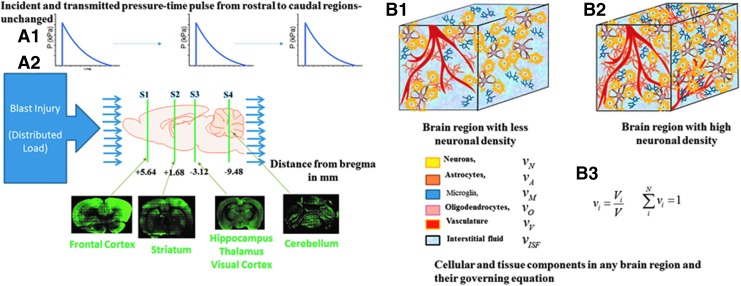
Schematic of experimental blast injury model and effective loading and tissue-specific response. (**A**) The top panel shows the blast overpressure-time pulse applied to the rostral to caudal to rostral regions of the rodent brain travels with minimal change because of very short duration of less than a millisecond. The bottom panel shows different regions S1 to S4 analyzed in this study (**B**) A given brain volume comprises the six components (neurons, astrocytes, microglia, oligodendrocyte, vasculature, and interstitial/cerebrospinal fluid) that can vary from region to region. The mechanical properties of the representative volume given in B3 indicate that the effective mechanical stresses in the volume and hence the neural components will be determined by the differential volume fraction of that cell type. The different nicotinamide adenine dinucleotide phosphate oxidase expressions seen in this work are hypothesized to be driven by this differential cell volume fraction. Color image is available online at www.liebertpub.com.neu

The precise mechanism by which bTBI increases NOX isoform expression is not known. A shock-wave passage throughout the brain during a transient period could initiate a mechanical disturbance to plasma membrane structures within the brain parenchyma. It is reasonable, considering the small size of the rat brain and relatively thin skull, to assume the shockwave loading of the brain is uniform within brain structures investigated in this work (frontal cortex, striatum, hipocampus, and thalamus and cerebelllum). The uniformity of the pressure field within the rat brain under blast loading conditions was demonstrated in a numerical model^77^ and was validated via intracranial pressure measurements by our group (data not published). If this is true—i.e. the mechanical forces created by the shockwave are distributed uniformly, and considering the brain's highly anisotropic and heterogeneous organization—our data suggest the extent of the local damage would depend on the composition and microarchitecture of a specific brain region (Fig. 10).

## Conclusion

Our studies demonstrate that moderate blast injury causes an increase in NOX isoforms in various brain regions to a differential degree. Increased NOX isoforms correlated with a concomitant rise in superoxide levels in corresponding regions that show a higher increase in NOX expression indicating oxidative damage. In addition, protein oxidation product 4HNE showed a strong tendency to increase in different brain regions. Further, higher increase in NOX isoforms in neurons compared with other brain cells strongly suggests neurons are by far the most vulnerable to oxidative damage in the early evolution of injury pathology.
